# Implementing Wastewater-Based Epidemiology for Long-Read Metagenomic Sequencing of Antimicrobial Resistance in Kampala, Uganda

**DOI:** 10.3390/microorganisms13061240

**Published:** 2025-05-28

**Authors:** William Strike, Temitope O. C. Faleye, Brian Lubega, Alexus Rockward, Soroosh Torabi, Anni Noble, Mohammad Dehghan Banadaki, James Keck, Henry Mugerwa, Matthew Scotch, Scott Berry

**Affiliations:** 1 Department of Biomedical Engineering, University of Kentucky, 522 Robotics and Manufacturing Building, Lexington, KY 40506, USA; dalton.strike@uky.edu (W.S.); arockward@uky.edu (A.R.); 2 Biodesign Center for Environmental Health Engineering, Arizona State University, 1001 S McAllister Ave, Tempe, AZ 85281, USA; tfaleye@asu.edu (T.O.C.F.); matthew.scotch@asu.edu (M.S.); 3 Joint Clinical Research Centre, Lubowa Estates, Kampala 759125, Uganda; blubega@jcrc.org.ug (B.L.); jcrc@jcrc.org.ug (H.M.); 4 Department of Mechanical and Aerospace Engineering, University of Kentucky, 151 Ralph G. Anderson Building, Lexington, KY 40506, USA; s.torabi@uky.edu (S.T.); anni.noble@uky.edu (A.N.); mdehghan94@uky.edu (M.D.B.); 5 WWAMI School of Medicine, University of Alaska Anchorage, 3211 Providence Dr, Anchorage, AK 99508, USA; jwkeck@anthc.org; 6 College of Health Solutions, Arizona State University, 550 N. 3rd Street, Phoenix, AZ 85281, USA

**Keywords:** wastewater, metagenomic environmental surveillance, drug-resistant pathogens

## Abstract

Antimicrobial resistance (AMR) is an emerging global threat that is expanding in many areas of the world. Wastewater-based epidemiology (WBE) is uniquely suited for use in areas of the world where clinical surveillance is limited or logistically slow to identify emerging threats, such as in Sub-Saharan Africa (SSA). Wastewater was analyzed from three urban areas of Kampala, including a local HIV research clinic and two informal settlements. Wastewater extraction was performed using a low-cost, magnetic bead-based protocol that minimizes consumable plastic consumption followed by sequencing on the Oxford Nanopore Technology MinION platform. The majority of the analysis was performed using cloud-based services to identify AMR biomarkers and bacterial pathogens. Assemblies containing AMR pathogens were isolated from all locations. As one example, clinically relevant AMR biomarkers for multiple drug classes were found within *Acinetobacter baumannii* genomic fragments. This work presents a metagenomic WBE workflow that is compatible with areas of the world without robust water treatment infrastructure. This study was able to identify various bacterial pathogens and AMR biomarkers without shipping water samples internationally or relying on complex concentration methods. Due to the time-dependent nature of wastewater surveillance data, this work involved cross-training researchers in Uganda to collect and analyze wastewater for future efforts in public health development.

## 1. Introduction

In 2019, approximately 1.27 million deaths were attributable to drug-resistant bacteria globally [1]. We are faced with a future where common bacterial infections will require more extensive AMR diagnostics or multiple treatments, adding thousands of dollars in direct healthcare costs to treat a single patient [2,3]. Rural and underserved regions of the world with less access to healthcare are more likely to be prescribed broad-spectrum antibiotics on initial appointments [4,5]. Therefore, there are major antibiotic stewardship concerns in Sub-Saharan Africa (SSA) that are impacted by diagnostic cost, healthcare capability, and lack of routine pathogen surveillance [6]. Kampala, Uganda, is one of the fastest growing cities worldwide due to economic growth in the region and refugee intake from nearby conflicts [7]. Approximately 10% of Kampala residents are connected to the sewer system, with the remaining population relying on pit-latrines, straight pipes, or septic tanks. Kampala is surrounded by large wetlands and discharges multiple wastewater sources into nearby Lake Victoria, which functions as a water reservoir for industrial practices and agricultural irrigation [8]. Only 43% of urban Uganda residents have access to safely managed water sources, and the majority have limited sanitation services [9,10]. This area is prone to diarrheal diseases, such as drug-resistant and drug-sensitive *Vibrio cholerae*, *Escherichia coli*, and *Salmonella enterica*, which are often spread through waste-contaminated drinking water [11,12,13]. Gastrointestinal diseases are of great concern for people living with HIV/AIDS (PLWHA) who are at a higher risk of hospital-acquired infections (HAIs). Although Uganda has made notable progress in combatting HIV and HAIs, there are still major concerns for AMR accumulation in the local water system that may interact with the public. Drug-susceptible HAI surveillance can be performed using culture-based methods, 16S sequencing, or targeted polymerase chain reaction. However, in the context of patients living with HIV in Uganda, high prescription rates of antimicrobials and lowered immune system functionality increase the risk of drug-resistant co-infections and may complicate HIV treatment [14,15]. This work presents a novel workflow combining low-cost nucleic acid extraction and comparably low-cost DNA sequencing for use in wastewater AMR surveillance in Kampala, Uganda.

Wastewater may serve as a “perfect storm” for antimicrobial resistance gene (ARG) proliferation due to constant selective pressure from antibiotics, agricultural runoff, and industrial practices at certain stages of wastewater treatment [16]. Therefore, there is a need to identify AMR pathogens and mobile genetic units (MGEs) carrying clinically relevant biomarkers in local water systems. This study builds on a reported nucleic acid extraction workflow that reduces labor and consumable cost considerably; however, metagenomic sequencing costs remain prohibitive in low–middle-income countries (LMICs) [6,17,18,19].

## 2. Materials and Methods

Three 250 mL wastewater grab samples were collected and analyzed in this study. Three sampling sites were visited on the 10th and 11th of May 2023 in Kampala, Uganda: Joint Clinical Research Centre (JCRC) (sample 1), Kawaala (sample 2), and Mulago neighborhoods (sample 3). On the JCRC campus, the sample was collected from a septic tank downstream of the clinic building. From Mulago and Kawaala, the samples were taken from the central flow of open-air sewage channels that eventually drain into Lake Victoria. This preliminary work represents a single time point and does not represent long-term surveillance in Kampala.

All samples were transported on ice to a class 2 biosafety laboratory at JCRC for sample processing. Each sample (250 mL) was filtered using 0.45 μm cellulose acetate and cellulose nitrate filters from the MILLICUP™-FLEX Disposable Filtration Kit (MilliporeSigma, Darmstadt, Germany). Filter-trapped solids were resuspended in 625 µL of lysis buffer (4 M guanidinium thiocyanate (GTC), 0.01 4-morpholinethanesulfonic acid pH = 5.5, dissolved in 50% ethanol) and subjected to nucleic acid extraction by bead bashing and our previously reported Exclusion-based Sample Preparation (ESP) method where analyte-bound paramagnetic beads are manipulated through wash buffers [19]. Two replicates of 625 µL of suspended filter-trapped solids were processed as described elsewhere with slight modifications to filtration and AMPure bead cleanup [19,20]. Briefly, the samples were mixed with 10 µL each of 400 nm and 700 nm diameter paramagnetic particles (SeraSil-Mag™, Cytiva Life Sciences, Marlborough, MA, USA) heated at 50 °C and vortexed every 5 min, for a total of 20 min, and then placed in a hula mixer at ambient temperature for another 20 min. Next, a magnetic rack and pipette were used to remove the lysis buffer from the analyte-bound beads followed by resuspension in wash buffer 1 (1 M GTC, 10 mM Tris pH = 8.0, 0.01% Tween-20). The resuspended beads were then transferred to an ESP plate (22100008, Gilson, Madison, WI, USA), where they were thoroughly and sequentially mixed in wells of fresh wash buffer 1 and wash buffer 2 (10 mM Tris ph = 8.0 in 80% Ethanol). Finally, the beads were mixed with 100 µL of nuclease-free water and heated at 70 °C for another 20 min to aid the elution of the nucleic acids from the beads. The eluate was then transferred into a 1.5 mL tube for storage at −80 °C. Nucleic acid extract concentrations were quantified via the Qubit™ dsDNA Quantification Assay Kit (Invitrogen, Waltham, MA, USA) on a Qubit 3.0 instrument before the post-extraction cleanup. AMPure XP beads (A63880, Beckman Coulter, Blair, CA, USA) were mixed at 1.8× sample volume, rotated at room temperature for 10 min, and placed on a magnetic rack until the solution was clear. Next, the solution was removed using a pipette and the beads were bathed in two separate washes of 200 µL of 70% ethanol. After removing the final ethanol wash solution, the tube cap was left open to air-dry the beads for at least 30 s. The dry beads were then eluted in 12 µL of nuclease-free water and incubated for 10 min at room temperature. The sample was then placed on a magnetic rack and the eluate was transferred to a new 1.5 mL tube.

Library preparation was performed using the Oxford Nanopore Native Barcoding Kit V14 (SQK-NBD114.24, Oxford Nanopore Technologies, Oxford, UK) following the ligation sequencing gDNA protocol. Briefly, 400 ng of DNA from each extract underwent end-prep repair followed by barcode ligation, equimolar pooling, adapter ligation, and sequencing on an R.10 MinION (Oxford Nanopore Technologies, Oxford, UK) flow cell. Base-calling was performed using Guppy version 6.5.7 with GPU acceleration using the DNA r10.4.1 e8.2 400 bps high-accuracy model.

Reads were trimmed, assembled, and polished using Porechop, Flye, and Medaka, respectively, as implemented in Nanogalaxy [21]. Taxonomic identification was performed using the Epi2me Agent Fastq What’s In My Pot (WIMP). Taxonomic heatmaps are represented by dividing the number of reads identified per top ten most common genera by the total number of reads at each location. Subsequently, contigs were queried against multiple ARG databases using ABRicate to identify ARG encoding contigs. These contigs were then used as query in a BLASTn search of the GenBank database, and the top two hits were documented. The polished assemblies were also fed into the Resistance Gene Identifier (RGI) version 6.0.3 software to compare ARGs between sampling location [22]. Raw data are available upon request.

## 3. Results

The sequencing metrics are shown in Table 1. The raw and processed reads per sampling location ranged from 114,934 bp to 368,007 bp and 105,114 bp to 337,838 bp, respectively. Contigs recovered per sampling location, length, and depth of coverage ranged from 101 to 798, 108 bp to 1,322,077 bp, and 3× to 1924×, respectively (Table 1). It is important to note that 63.4% (798/1259) of the contigs recovered in this study were from sample 1.

**Figure 1:**
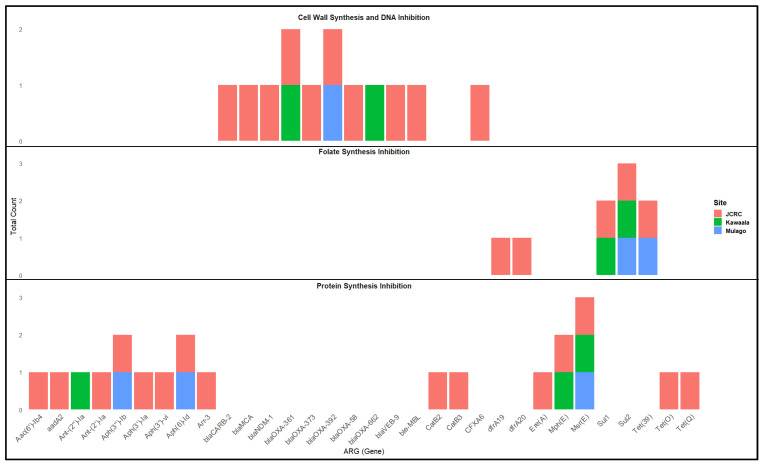
ABRicate-identified ARGs at each sampling location separated by mechanism of action.

In all, 36 ARGs were detected (Figure 1). However, the ARGs detected per sample ranged from 7 to 31 and the ARG-bearing contigs detected per sample ranged from 3 to 18. The length of the ARG-bearing contigs, depth of coverage, and number of ARGs per contig ranged from 923 bp to 941,881 bp, 4× to 22× and 1–9, respectively (Table 1). Three distinct *sul1* genes were identified in samples 1 and 2 (Tables S2 and S3); however, this is only represented in a single binary detection in Figure 1.

**Figure 2:**
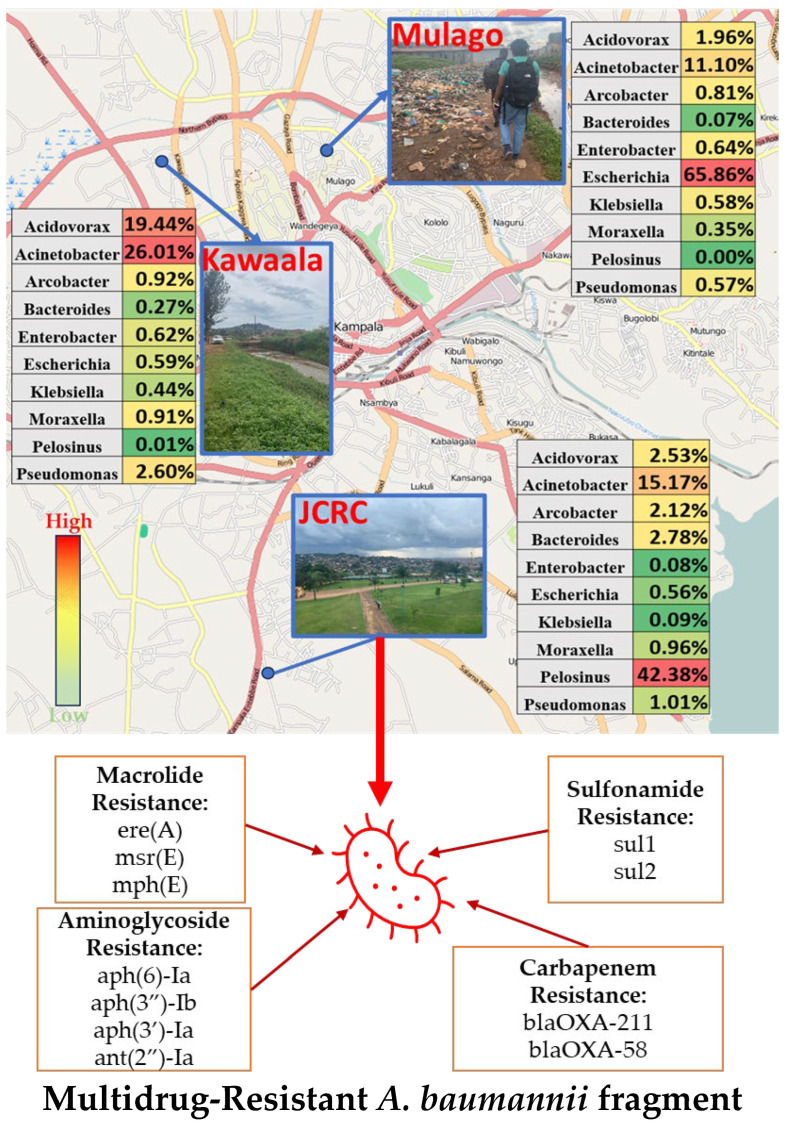
Metagenomic analysis of wastewater in Kampala, Uganda, showing relative percentage of the top ten bacterial genera (concentration gradient where high concentration is represented by red and low concentration is represented by green); bottom: multidrug-resistant *A. baumannii* assembly fragment identified from JCRC wastewater.

Most of our clinically relevant AMR pathogen reads belonged to the *Acinetobacter* genus, a highly virulent Gram-negative bacterium that is mostly associated with nosocomial infections (Figure 2, Table 2) [23]. Multidrug-resistant *A. baumannii* has been reported in clinical isolates in Kampala, Uganda [24]. However, there is a lack of knowledge on how drug-resistant *A. baumannii* is disseminated into the environment through wastewater in this region.

The 36 ARGs detected in this study have been shown to confer resistance to antibiotics with varied targets and mechanisms of action. Specifically, the targets include cell wall, protein, and folate synthesis (Figure 1, Tables S1–S4). Only 2% (25/1259) of the contigs detected from WW in this study had ARGs encoded, and 72% (18/25) of these ARG-bearing contigs were from sample 1. Precisely, 48% (12/25) of the contigs encoded more than one ARG and the three contigs encoding the highest number of ARGs (6, 8, and 8) were recovered from sample 1 (Table S2).

Each sampling location was taxonomically diverse using WIMP analysis via Epi2me Agent with Fischer’s alpha above 220 for all locations (Figure 2). Many of the high-abundance genera identified in our unassembled data are common wastewater bacteria not normally known to cause disease (*Pelosinus*, *Acidovorax*, *Bacteroides*). However, after assembly, many of the clinically relevant biomarkers were related to pathogenic bacteria, including various *Acinetobacter* species (Figure 2, Table 2).

## 4. Discussion

Contigs from site 1 (Table 2 and Table S2) were most closely mapped to *Acinetobacter* plasmids containing various ARGs. Certain contigs from site 1 contained both macrolide resistance genes *msrE* and *mphE*, which are often reported together in mobile *Acinetobacter* plasmid-dif modules [25]. Additionally, this contig contained an extended spectrum beta lactamase *bla_CARB-2_* and sulfonamide resistance genes (*sul1*, *sul2*), which are associated with highly virulent *A. baumannii* mobile strains possessing *int1* [26,27,28]. The dataset presented suggests that ARGs present for multiple classes of antimicrobials are circulating within MGEs in the wastewater system. Contigs from site 1 containing two aminoglycoside resistance genes, *Aph(3″)-lb* and *Aph(6)-id*, varied in size dramatically (4030 bp–41,711 bp) but were also most closely identified to *Acinetobacter* plasmids (Table 2). Both of these phosphotransferase-encoding genes are commonly reported together in clinical isolates and MGEs [28,29,30].

This pathogen can cause a variety of illnesses, including skin infections, bacterial pneumonia, and bacteremia. Extensive drug-resistant *A. baumannii* is a growing concern due to poor antibiotic stewardship in hospital settings, especially during the SARS-CoV-2 pandemic [31]. Secondary and superinfections of *A. baumannii* in SARS-CoV-2-infected patients were reported in 2.4% of hospitalized patients in a recent study in Spain and were found to be the strongest determinant of mortality in ICU patients [32]. Third-generation and “last line” antibiotics are often required for the treatment of AMR isolates of *A. baumannii,* which may be in short supply or prohibitively expensive in LMICs. Previous studies have reported carbapenem-resistant *A. baumannii* in clinical and environmental samples near our sampling point in Mulago [33,34].

*A. baumannii* acquires drug resistance to a broad range of antibiotics (including broad-spectrum β-lactams, aminoglycosides, and fluoroquinolones) through a plethora of mechanisms described in detail elsewhere [31]. Many of our *Acinetobacter* reads were further classified to the less pathogenic *johnsonii* species; however, a recent study has found that *A. johnsonii* is capable of harboring multiple clinically relevant ARGs and spreading them to other *Acinetobacter* species via lytic bacteriophages [35]. The sequencing of *A. baumannii* isolates has associated certain integrase genes (*intI1*, *intI2*) with epidemic potential and multidrug resistance [27]. Drug-resistant *A. baumannii* is of great concern for hospitals, with some risk assessment programs requiring as few as two cases of drug-resistant *A. baumannii* infection to trigger active surveillance and internal control measures [36]. This quick reactionary plan is likely a response to the rising prevalence of carbapenem-resistant *A. baumannii* and increased mortality rate in infected immunosuppressed patients [37]. Although treatments related to certain immunosuppressive diseases (such as HIV) have improved, there are major opportunistic infection concerns during and after treatment.

A contig from site 2 was most closely mapped to a biosolids bacterium plasmid (MN366358.1) related to ARG transfer in Salmonella typhimurium [38]. This study identified three of the four main ARGs (Mph(E), Msr(E), Sul1) published in the original isolation from Law et al. (2021) [38]. A separate contig from site 2 most closely resembled Acinetobacter cumulans and contained multiple ARGs: Msr(E), Mph(E), Aph(3″)-lb, Aph(6)-id, and Sul2. This contig is significant because it was assembled from a grab sample located near the Mulago hospital and high-density slums. The largest contig from site 2 (58,121 bp) also closely matched to Acinetobacter and contained our only identification of carbapenem-resistant gene bla_OXA-643_ (Figure 1, Table S4). Bacterial infections accounted for around 20% of patient deaths in Uganda between 2014 and 2015 and 25% of child deaths within the same period. The majority of clinical isolates taken during this previous study were found to be resistant to first-line antibiotics, but only 5% were resistant to newer or locally unavailable antibiotics such as gentamicin [39]. Unfortunately, AMR prevalence has rapidly increased in a relatively short period of time in Uganda, and more recent studies have isolated Escherichia coli, K. pneumoniae, and Staphylococcus aureus with high gentamicin resistance rates (51.8–83.2%) [13,40]. Our identification of aminoglycoside resistance genes (Aph(3″)-lb and Aph(6)-id) also suggests that the wastewater environment may be a reservoir for gentamicin-resistant organisms (Figure 1, Tables S1–S4).

Standard methods to isolate clinical samples for gentamicin-resistant bacteria may take 2–3 days, assuming that the hospital has the available staff, space, and reagents necessary to perform the assay. Current methods for identifying AMR biomarkers or known HAI organisms in the environment generally rely on PCR or phenotypic culture assays. These methods are either highly tailored to specific pathogens and resistance genes or require extensive time to isolate certain organisms. Many of these assays are relatively complex to perform and require larger laboratory space for routine surveillance. This work suggests that metagenomic WBE may supplement other surveillance networks in low-resource settings to identify which resistant and non-resistant pathogens are circulating within the local environment. Almost all antibiotics or antibiotic metabolites are excreted in urine or feces, which can accumulate in the wastewater system and affect the local environment [16]. LMICs consume antibiotics at a higher rate due to less regulations and higher incidence of infectious disease, which is of great concern for immunosuppressed individuals such as PLWHA [41]. Although mortality due to HIV/AIDS in Uganda has decreased rapidly over the last few decades, PLWHA are approximately seven times more likely to develop bacterial pneumonia and bacteriuria than HIV sero-negative patients [14,15]. Therefore, major cities in LMICs without sufficient wastewater treatment processes are at a higher risk of environmental infections and may benefit most from wastewater surveillance. For example, cholera outbreaks are directly caused by contaminated public water systems in Kampala, which require extensive surveillance efforts to combat [42]. Urban LMIC wastewater may interact with the populace through direct contact or through agricultural practices. Urban agriculture accounts for approximately 90% of perishable vegetables through cultivation along roadsides, rivers, canals, or parks [43]. This industry may not be sanctioned by landowners or the local government but appears to be well tolerated. These spaces are generally used by migrants who compete for unused irrigated sites, especially during dry seasons [43]. A 2000 report found that a third of households in Kampala participate to some degree in urban agriculture to offset food security concerns [44]. The majority of commercial and household urban farmers in Kampala are women, which may be due to social and economic expectations in Uganda [44]. Urban farmers are forced to rely on untreated wastewater irrigation due to water scarcity, which may recirculate water-borne pathogens into the food chain [41,43].

Long-read metagenomic wastewater data are generally scarcer in some sequencing metrics (e.g., quality score, coverage, depth) compared to short-read Illumina data. However, this study relied on the Oxford nanopore sequencing platform due to cost and sustainability concerns in SSA. As an aside, the Ugandan laboratory where nucleic acid extraction was performed had access to high-end Illumina sequencing equipment but was unable to rely on this platform for other projects due to maintenance issues and reagent availability in the region. Although significant efforts to reduce the cost of WBE through simplified nucleic acid extraction techniques have succeeded, sequencing costs are still prohibitive in SSA. Unfortunately, this study was unable to generate full genomes out of metagenomic data, and future work is needed to phenotypically validate drug resistance through targeted sequencing or culture studies. Therefore, there is a need to increase the sequencing throughput of pathogenic organisms to increase coverage and depth of downstream AMR and taxonomic analysis. Additionally, this work describes three grab samples at a single time point due to the logistic difficulty of surveillance in SSA and exploratory nature of this study. Future work is needed to better understand temporal variations in surface water metagenomic surveillance in this region. Bacterial diversity and concentration are subject to change over time due to changes in temperature, rainfall, etc. Therefore, a single sampling point will not be representative of the region over time. Additionally, our filter-based preconcentration method may not be appropriate for studies interested in surveilling spore-forming bacteria or viral particles that may pass through the 0.45 µm filter. The previously described low-cost sample preparation method is reported to reduce nucleic degradation [20], but it remains to be studied how open-air canals in high-density SSA chemically differ from wastewater found in our previous work, which may affect extraction or sequencing efficiency. Additionally, this study only represents a single time point. Longer longitudinal studies are necessary to better understand AMR dissemination within the region.

## 5. Conclusions

There are unique challenges and opportunities for metagenomic WBE in SSA. A lack of access to wastewater infrastructure may allow for pathogens harboring ARGs to proliferate within the environment and contaminate drinking wells or urban agriculture. Limited wastewater infrastructure may be contributing to the recirculation of AMR biomarkers from clinical settings into the environment and food chain. Current methods for WBE or large-scale public health surveillance rely on reoccurring shipments of samples to out-of-country institutions or bioanalytical companies, which are expensive, prone to sample degradation, and it takes multiple days to communicate their findings. However, this work presents a “flipped-model” where local scientists can be trained to collect and manage their own data, which would eliminate degradation due to shipping, reduce costs, and build in-country expertise on cutting-edge molecular epidemiological techniques. Therefore, metagenomic WBE may be a highly cost-efficient method to identify circulating pathogens and AMR biomarkers at facility and/or population scales. This study successfully developed and implemented a wastewater based metagenomic workflow that is compatible with the unique challenges of SSA. This study identified various clinically relevant AMR biomarkers and opportunistic pathogens at a research clinic servicing PLWHIV while reducing cost per sample. At a local clinic, the genomic fragments of *A. baumannii* with ARGs related to various clinically relevant drug classes were identified. Additionally, our pathogen assemblies sampled from open-air canals in densely populated informal settlements are of great concern since many people use this water for urban agriculture. This work suggests that relatively low-utility extraction and analysis techniques may support public health efforts at the building or city level and may alert healthcare officials to oncoming drug-resistant outbreaks.

## Figures and Tables

**Table 1:** Experimental sequencing metrics; # = number.

S/N	Site	Gbs of Data	#Reads	#Trimmed Reads	#Contigs	Range Mean Depth	Range Contig Length	#ARGs	ARG-Bearing Contigs #	Range Mean Depth	Range Contig Length	Range #ARGs/ Contig
S1-J	JCRC	0.918	368,007	337,838	798	3×–919×	296 bp–511,847 bp	31	18	4×–22×	923 bp–90,589 bp	1–9
S2-K	KAWAALA	0.289	114,934	105,114	101	3×–1924×	108 bp–941,881 bp	7	4	5×–12×	8006 bp–941,881 bp	1–4
S3-M	MULAGO	0.832	268,486	245,787	360	3×–748×	508 bp–1,322,077 bp	7	3	14×–18×	6358 bp–58,121 bp	1–5

**Table 2:** Top 2 GenBank hits of the contigs encoding ARGs. %SIML = percentage similarity; # = number; * denotes genomic reference database outside of blast core_nt.

		Contig-ID	Contig Length (bp)	Mean Depth of Coverage	Accession #	Query Cover	%Siml	Organism	Accession #	Query Cover	%Siml	Organism
1	S1-J	Contig_37	44,316	22	CP096936.1	17	92.94	*Pseudomonas aeruginosa* plasmid	CP028568.2	17	92.95	*Aeromonas hydrophila* chromosome
2		Contig_44	923	7	CP053365.1	100	99.57	*Klebsiella pneumoniae* plasmid	LC537594.1	100	99.57	*Acinetobacter lwoffii* plasmid
3		Contig_61	31,822	19	CP051867.1	83	99.94	*Acinetobacter baumannii* plasmid	CP028560.1	57	99.82	*Acinetobacter* sp. plasmid
4		Contig_62	41,771	13	LC591943.1	75	99.15	*Acinetobacter variabilis* plasmid	CP038646.1	75	99.28	*Acinetobacter baumannii* plasmid
5		Contig_65	4030	22	CP051867.1	100	99.90	*Acinetobacter baumannii* plasmid	CP079749.1	98	99.67	*Acinetobacter johnsonii* plasmid
6		Contig_79	4549	20	CP051867.1	100	98.82	*Acinetobacter baumannii* plasmid	AJ605332.1	67	98.47	*Pasteurella multocida* ARGs dfrA20 and sul2
7		Contig_97	4081	7	CP132737.1	41	99.52	*Klebsiella pneumoniae* plasmid	CP132632.1	41	99.52	*Klebsiella pneumoniae* plasmid
8		Contig_103	35,592	11	CP104579.1	27	99.30	*Pseudomonas oleovorans* chromosome	LR698992.1	25	99.89	*Laribacter hongkongensis* chromosome
9		Contig_116	7031	13	CP051867.1	98	99.89	*Acinetobacter baumannii* plasmid	CP115643.1	50	99.86	*Acinetobacter baumannii* chromosome
10		Contig_154	90,589	8	CP042464.1	96	95.83	*Segatella copri* chromosome	CP102288.1	90	95.68	*Segatella copri* chromosome
11		Contig_160	5510	20	CP042557.1	74	98.24	*Acinetobacter baumannii* plasmid	MK413719.1	60	98.24	*Klebsiella pneumoniae* plasmid
12		Contig_163	23,722	4	CP019041.1	84	98.52	*Acinetobacter junii* chromosome	CP090382.1	99	97.63	*Acinetobacter towneri* chromosome
13		Contig_204	56,173	18	CP055277.1	99	96.82	*Acinetobacter* sp. chromosome	CP090416.1	98	96.54	*Acinetobacter johnsonii* chromosome
14		Contig_236	9742	8	CP104863.1	33	97.67	*Stenotrophomonas maltophilia* chromosome	MN366358.1	33	97.64	Bacterium plasmid
15		Contig_250	29,575	6	AP025941.1	62	93.79	*Paraprevotella clara* genome	CP120849.1	66	95.81	*Parabacteroides chongii* chromosome
16		Contig_340	1042	17	MT011984.1	100	97.90	*Comamonas testosteroni* plasmid	MK638972.1	100	97.90	*Escherichia coli* plasmid
17		Contig_388	11,301	6	CP100430.1	59	98.62	*Streptococcus suis* chromosome	LR698970.1	59	98.60	*Lachnospiraceae bacterium* chromosome
18		Contig_880	11,299	15	CP033122.1	88	97.50	*Acinetobacter wuhouensis* plasmid	CP032277.1	75	97.14	*Acinetobacter* sp. plasmid
19	S2-K	Contig_105	8006	5	MN366358.1	93	98.62	Bacterium plasmid	CP083711.1	76	98.81	*Enterobacter cloacae* complex sp. plasmid
20		Contig_29	941,881	12	* GTDB-Tk-v1.7.0 GCF_000368045.1	94	96.3	*Acinetobacter johnsonii*				
21		Contig_34	8025	5	CP053947.1	100	92.17	*Acinetobacter* sp.	LC537318.1	100	92.16	*Acinetobacter lwoffii*
22		Contig_39	105,337	7	CP045051.1	65	93.56	*Acinetobacter johnsonii* chromosome	CP065666.1	69	94.90	*Acinetobacter johnsonii* chromosome
23	S3-M	Contig_27	58,121	16	CP090180.1	93	97.75	*Acinetobacter johnsonii* chromosome	CP031011.1	92	97.71	*Acinetobacter johnsonii* chromosome
24		Contig_35	14,796	18	CP035937.1	67	99.97	*Acinetobacter cumulans* plasmid	CP053947.1	29	100	*Acinetobacter* sp. WY4 chromosome
25		Contig_79	6358	14	CP065052.1	45	99.27	*Acinetobacter baumannii* plasmid	CP053947.1	45	99.17	*Acinetobacter* sp. WY4 chromosome

## Data Availability

Data are available upon request.

## Supplementary Materials

The following supporting information can be downloaded at: https://www.mdpi.com/article/10.3390/microorganisms13061240/s1, Table S1: RGI derived qualitative table of ARGs identified at each sampling location where “+” represents a perfect hit, “/” represents a strict hit, and “-“ represents a no or low detection; Table S2: ARGs identified in each assembled contig containing at least one ARG (denoted by a red cell) separated by mechanism at site 1: JCRC; Table S3: ARGs identified in each assembled contig containing at least one ARG (denoted by a red cell) separated by mechanism at site 2: Kawaala; Table S4: ARGs identified in each assembled contig containing at least one ARG (denoted by a red cell) separated by mechanism at site 3: Mulago.

## Abbreviations

The following abbreviations are used in this manuscript:

LMICs	Low- and middle-income countries
SSA	Sub-Saharan Africa
WBE	Wastewater-based epidemiology
AMR	Antimicrobial resistance
MGE	Mobile genetic units
ESP	Exclusion-based sample preparation
RGI	Resistance Gene Identifier
WIMP	What’s In My Pot
PLWHA	People living with HIV/AIDS
