# Supplementary material for: Implementing Wastewater-Based Epidemiology for Long-Read Metagenomic Sequencing of Antimicrobial Resistance in Kampala, Uganda
Source: Microorganisms. 2025 May 28;13(6):1240. doi: 10.3390/microorganisms13061240 (PMC12195534; doi:10.3390/microorganisms13061240)
Supplement: Supplementary file 1 [file microorganisms-13-01240-s001.zip › microorganisms-3642910-supplementary.pdf]

Implementing wastewater-based epidemiology for long-read metagenomic sequencing of antimicrobial resistance in Kampala, Uganda

Supplemental Information

Each polished assembly was analyzed via the RGI bioinformatic tool through the Comprehensive Antibiotic Resistance Database (CARD) to compare ARGs across each sampling point (Table S1). A perfect RGI match is identical along the entire CARD reference sequence. A strict RGI match is a partial hit with a bit-score of at least 500. A total of 46 ARGs were identified in the CARD database with a majority of the strict and perfect ARG hits within the JCRC sample which may be due to the septic tank sampling infrastructure in comparison to a more dilute open-air canal. Additionally, all 5 of the strict hits from the JCRC sample were also identified in the Mulago sample, and 1/5 (*Sul2*) was found in all 3 sampling locations. ABRicate was used to query polishes contigs against multiple ARG databases. Multiple ARGs were identified at each location (Table S2-S4).

Table S1: RGI derived qualitative table of ARGs identified at each sampling location where "**+**" represents a perfect hit, "**/**" represents a strict hit, and "**-**" represents a no or low detection.

JCRC	Kawaala	Mulago	ARG from CARD database
/	-	-	MexB
/	-	-	adeJ
/	-	-	cmeB
+	-	-	mtrE
/	-	+	qacEdelta1
/	/	/	qacG
/	-	-	qacJ
/	/	/	rsmA
/	-	/	tet(39)
/	-	-	AAC(6')-II
/	-	/	APH(3")-Ib
/	-	/	APH(6)-Id
/	-	-	GES-40
/	-	-	Mrx
/	-	-	OXA-211
/	-	-	OXA-309
/	-	-	OXA-373
/	-	-	OXA-498
/	-	/	OXA-644
/	-	-	OXA-645
/	-	-	OXA-650
/	-	-	OXA-651
/	-	-	OXA-652
/	-	-	OXA-895
/	-	-	PNGM-1
/	-	-	SAT-3
/	-	-	aadA17
/	-	-	aadA2
/	-	-	aadA22
/	-	-	aadA23
/	-	-	aadA25
/	-	-	apmA
/	-	-	Mph(A)
+	-	+	Mph(E)
/	-	-	ErmN
/	-	-	*E. coli* EF-Tu mutants conferring resistance to Pulvomycin
/	-	-	RlmA(II)
/	-	-	*S. aureus* mupB conferring resistance to mupirocin
/	-	-	eptB
/	-	-	vanK gene in vanl cluster
/	-	-	vanR gene in vanL cluster
/	/	/	vanW gene in vanG cluster
+	-	+	Msr(E)
/	-	-	mecI
+	-	+	sul1
+	+	+	sul2
**Sampling Site**			

Table S2: ARGs identified in each assembled contig containing at least one ARG (denoted by a red cell) separated by mechanism at site 1: JCRC.

	Cell wall synthesis and DNA inhibition												Protein synthesis inhibition																	Folate synthesis						
**Contig #**	ble-MBL	CFXA6	blaOXA-392	blaMCA	blaCARB-2	blaOXA-643	blaOXA-58	blaOXA-373	blaNDM-1	blaOXA-662	blaOXA-361	blaVEB-9	Aph (3') -vi	CatB3	CatB2	Aac (6')-Ib4	Ant-(2')-Ia	Ant-(2'')-Ia	Aph(3')-Ia	Mph(E)	Msr(E)	Ere(A)	Arr-3	Aph(3'')-Ib	aadA2	Aph(6)-Id	aadA2	Tet(Q)	Tet(O)	Tet(39)	Sul2	Sul1	Sul1	Sul1	dfrA19	dfrA20
**37**																				■	■															
**44**													■																							
**61**																				■	■			■		■				■	■					
**62**					■											■				■	■		■								■		■		■	
**65**																								■		■										
**79**																															■					■
**97**	■								■																											
**103**															■		■					■		■	■	■	■						■			
**116**							■										■		■																	
**154**		■																																		
**160**												■		■																			■			
**163**			■														■																			
**204**								■																												
**236**																																	■			
**250**																												■								
**340**																																■				
**388**																													■							
**880**				■																																

Table S3: ARGs identified in each assembled contig containing at least one ARG (denoted by a red cell) separated by mechanism at site 2: Kawaala.

	Cell wall synthesis and DNA inhibition												Protein synthesis inhibition																	Folate synthesis						
**Contig #**	ble-MBL	CFXA6	blaOXA-392	blaMCA	blaCARB-2	blaOXA-643	blaOXA-58	blaOXA-373	blaNDM-1	blaOXA-662	blaOXA-361	blaVEB-9	Aph (3') -vi	CatB3	CatB2	Aac (6')-Ib4	Ant-(2')-Ia	Ant-(2'')-Ia	Aph(3')-Ia	Mph(E)	Msr(E)	Ere(A)	Arr-3	Aph(3'')-Ib	aadA2	Aph(6)-Id	aadA2	Tet(Q)	Tet(O)	Tet(39)	Sul2	Sul1	Sul1	Sul1	dfrA19	dfrA20
**105**																		■		■	■													■		
**29**										■																										
**34**											■																									
**39**																															■					

Table S4: ARGs identified in each assembled contig containing at least one ARG (denoted by a red cell) separated by mechanism at site 3: Mulago.

	Cell wall synthesis and DNA inhibition												Protein synthesis inhibition																	Folate synthesis						
**Contig #**	ble-MBL	CFXA6	blaOXA-392	blaMCA	blaCARB-2	blaOXA-643	blaOXA-58	blaOXA-373	blaNDM-1	blaOXA-662	blaOXA-361	blaVEB-9	Aph (3') -vi	CatB3	CatB2	Aac (6')-Ib4	Ant-(2')-Ia	Ant-(2'')-Ia	Aph(3')-Ia	Mph(E)	Msr(E)	Ere(A)	Arr-3	Aph(3'')-Ib	aadA2	Aph(6)-Id	aadA2	Tet(Q)	Tet(O)	Tet(39)	Sul2	Sul1	Sul1	Sul1	dfrA19	dfrA20
**27**						■																														
**35**																					■			■		■					■					
**79**																														■						
